# Supplementary material for: RUNX2 isoform II protects cancer cells from ferroptosis and apoptosis by promoting PRDX2 expression in oral squamous cell carcinoma
Source: eLife. 2025 Jun 11;13:RP99122. doi: 10.7554/eLife.99122 (PMC12158427; doi:10.7554/eLife.99122)
Supplement: Figure 1—source data 1. [file elife-99122-fig1-data1.zip › Figure 1-Source Data/fig1-source data legends.docx]

**fig1-data1**. Original data corresponding to Figure 1B.

**fig1-data2**. Original data corresponding to Figure 1C.

**fig1-data3**. Original data corresponding to Figure 1D.

**fig1-data4**. Original data corresponding to Figure 1E.

**fig1-data5**. PDF file containing original RT-PCR images for Figure 1G, indicating the relevant bands and different OSCC patients.

**fig1-data6**. Original files for RT-PCR analysis displayed in Figure 1G.

**fig1-data7**. Original data corresponding to Figure 1H.

**fig1-data8**. Original data corresponding to Figure 1I.
