# Supplementary material for: RUNX2 isoform II protects cancer cells from ferroptosis and apoptosis by promoting PRDX2 expression in oral squamous cell carcinoma
Source: eLife. 2025 Jun 11;13:RP99122. doi: 10.7554/eLife.99122 (PMC12158427; doi:10.7554/eLife.99122)
Supplement: Figure 2—source data 1. [file elife-99122-fig2-data1.zip › Figure 2-Source Data/fig2-data3.pdf]

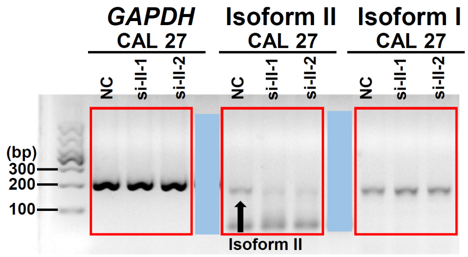


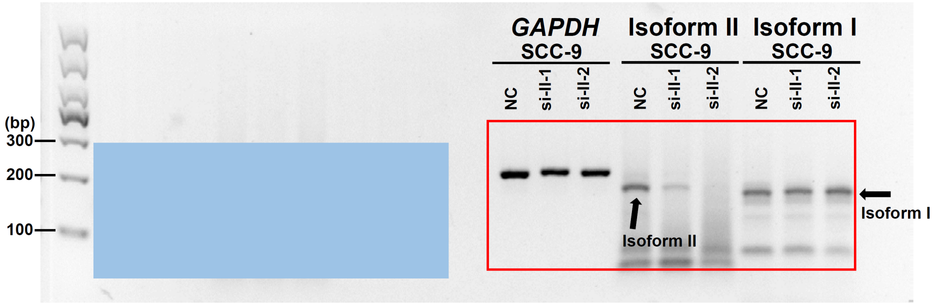


**Figure 2, Source Data 3.** Original RT-PCR images corresponding to Figure 2D. The upper or lower image corresponds to CAL 27 or SCC-9, respectively. *GAPDH* served as a loading control.
