# Supplementary material for: RUNX2 isoform II protects cancer cells from ferroptosis and apoptosis by promoting PRDX2 expression in oral squamous cell carcinoma
Source: eLife. 2025 Jun 11;13:RP99122. doi: 10.7554/eLife.99122 (PMC12158427; doi:10.7554/eLife.99122)
Supplement: Figure 2—source data 1. [file elife-99122-fig2-data1.zip › Figure 2-Source Data/fig2-source data legends.docx]

**fig2-data1**. PDF file containing original western blot images for Figure 2B, indicating the relevant bands and treatments.

**fig2-data2**. Original files for western blot analysis displayed in Figure 2B.

**fig2-data3**. PDF file containing original RT-PCR images for Figure 2D, indicating the relevant bands and treatments.

**fig2-data4**. Original files for RT-PCR analysis displayed in Figure 2D.

**fig2-data5**. Original data corresponding to Figure 2E.

**fig2-data6**. Original data corresponding to Figure 2F.

**fig2-data7**. Original data corresponding to Figure 2H.

**fig2-data8**. PDF file containing original RT-PCR image for Figure 2J, indicating the relevant bands and treatments.

**fig2-data9**. Original file for RT-PCR analysis displayed in Figure 2J.
