# Supplementary material for: RUNX2 isoform II protects cancer cells from ferroptosis and apoptosis by promoting PRDX2 expression in oral squamous cell carcinoma
Source: eLife. 2025 Jun 11;13:RP99122. doi: 10.7554/eLife.99122 (PMC12158427; doi:10.7554/eLife.99122)
Supplement: Figure 2—figure supplement 2—source data 1. [file elife-99122-fig2-figsupp2-data1.zip › Figure 2-figure supplement 2-Source Data/fig2-figsupp2-source data legends.docx]

**fig2-figsupp2-data1**. Original data corresponding to Figure 2-figure supplement 2A.

**fig2-figsupp2-data2**. Original data corresponding to Figure 2-figure supplement 2B.

**fig2-figsupp2-data3**. Original data corresponding to Figure 2-figure supplement 2C.
