# Supplementary material for: RUNX2 isoform II protects cancer cells from ferroptosis and apoptosis by promoting PRDX2 expression in oral squamous cell carcinoma
Source: eLife. 2025 Jun 11;13:RP99122. doi: 10.7554/eLife.99122 (PMC12158427; doi:10.7554/eLife.99122)
Supplement: Figure 3—source data 1. [file elife-99122-fig3-data1.zip › Figure 3-Source Data/fig3-source data legends.docx]

**fig3-data1**. Original data corresponding to Figure 3A.

**fig3-data2**. Original data corresponding to Figure 3B.

**fig3-data3**. Original data corresponding to Figure 3C.

**fig3-data4**. Original data corresponding to Figure 3D.

**fig3-data5**. Original data corresponding to Figure 3H.

**fig3-data6**. Original data corresponding to Figure 3I.

**fig3-data7**. Original data corresponding to Figure 3J.

**fig3-data8**. Original data corresponding to Figure 3K.

**fig3-data9**. Original data corresponding to Figure 3L.
