# Supplementary material for: RUNX2 isoform II protects cancer cells from ferroptosis and apoptosis by promoting PRDX2 expression in oral squamous cell carcinoma
Source: eLife. 2025 Jun 11;13:RP99122. doi: 10.7554/eLife.99122 (PMC12158427; doi:10.7554/eLife.99122)
Supplement: Figure 3—figure supplement 3—source data 1. [file elife-99122-fig3-figsupp3-data1.zip › Figure 3-figure supplement 3-Source Data/fig3-figsupp3-source data legends.docx]

**fig3-figsupp3-data1**. PDF file containing original RT-PCR image for Figure 3-figure supplement 3B, indicating the relevant bands and treatments.

**fig3-figsupp3-data2**. Original file for RT-PCR analysis displayed in Figure 3-figure supplement 3B.
