# Supplementary material for: RUNX2 isoform II protects cancer cells from ferroptosis and apoptosis by promoting PRDX2 expression in oral squamous cell carcinoma
Source: eLife. 2025 Jun 11;13:RP99122. doi: 10.7554/eLife.99122 (PMC12158427; doi:10.7554/eLife.99122)
Supplement: Figure 4—source data 1. [file elife-99122-fig4-data1.zip › Figure 4-Source Data/fig4-source data legends.docx]

**fig4-data1**. PDF file containing original RT-PCR images for Figure 4A, indicating the relevant bands and treatments.

**fig4-data2**. Original files for RT-PCR analysis displayed in Figure 4A.

**fig4-data3**. PDF file containing original RT-PCR images for Figure 4B, indicating the relevant bands and treatments.

**fig4-data4**. Original files for RT-PCR analysis displayed in Figure 4B.

**fig4-data5**. Original data corresponding to Figure 4B.

**fig4-data6**. PDF file containing original western blot images for Figure 4C, indicating the relevant bands and treatments.

**fig4-data7**. Original files for western blot analysis displayed in Figure 4C.

**fig4-data8**. Original data corresponding to Figure 4C.

**fig4-data9**. Original data corresponding to Figure 4D.

**fig4-data10**. PDF file containing original RT-PCR images for Figure 4E, indicating the relevant bands and treatments.

**fig4-data11**. Original files for RT-PCR analysis displayed in Figure 4E.

**fig4-data12**. Original data corresponding to Figure 4E.

**fig4-data13**. PDF file containing original western blot images for Figure 4F, indicating the relevant bands and treatments.

**fig4-data14**. Original files for western blot analysis displayed in Figure 4F.

**fig4-data15**. Original data corresponding to Figure 4F.

**fig4-data16**. Original data corresponding to Figure 4H.

**fig4-data17**. PDF file containing original western blot image for Figure 4I, indicating the relevant bands and treatments.

**fig4-data18**. Original file for western blot analysis displayed in Figure 4I.

**fig4-data19**. Original data corresponding to Figure 4J.

**fig4-data20**. Original data corresponding to Figure 4K.

**fig4-data21**. PDF file containing original western blot images for Figure 4L, indicating the relevant bands and treatments.

**fig4-data22**. Original files for western blot analysis displayed in Figure 4L.

**fig4-data23**. PDF file containing original RT-PCR image for Figure 4M, indicating the relevant bands and treatments.

**fig4-data24**. Original file for RT-PCR analysis displayed in Figure 4M.
