# Supplementary material for: RUNX2 isoform II protects cancer cells from ferroptosis and apoptosis by promoting PRDX2 expression in oral squamous cell carcinoma
Source: eLife. 2025 Jun 11;13:RP99122. doi: 10.7554/eLife.99122 (PMC12158427; doi:10.7554/eLife.99122)
Supplement: Figure 4—figure supplement 1—source data 1. [file elife-99122-fig4-figsupp1-data1.zip › Figure 4-figure supplement 1-Source Data/fig4-figsupp1-data4.pdf]

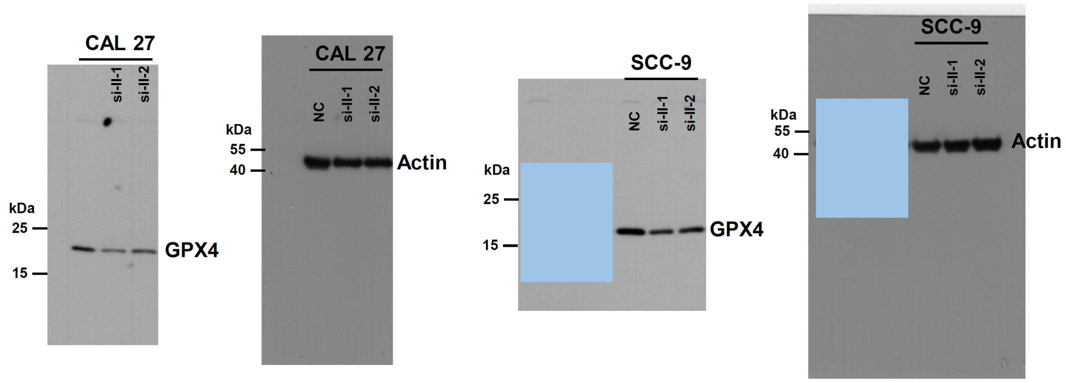

Figure 4-figure supplement 1, Source Data 4. Original Western blot images corresponding to Figure 4-figure supplement 1B. The two images on the left correspond to CAL 27. The two images on the right correspond to SCC-9. Actin served as a loading control.
