# Supplementary material for: RUNX2 isoform II protects cancer cells from ferroptosis and apoptosis by promoting PRDX2 expression in oral squamous cell carcinoma
Source: eLife. 2025 Jun 11;13:RP99122. doi: 10.7554/eLife.99122 (PMC12158427; doi:10.7554/eLife.99122)
Supplement: Figure 4—figure supplement 3—source data 1. [file elife-99122-fig4-figsupp3-data1.zip › Figure 4-figure supplement 3-Source Data/fig4-figsupp3-data1.pdf]

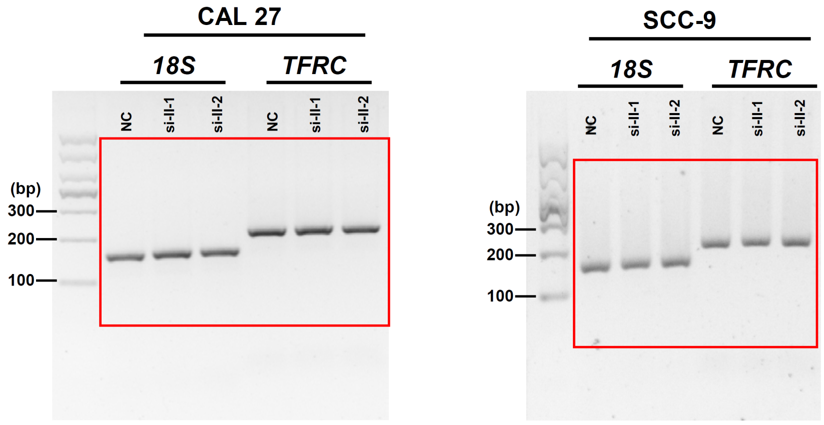

Figure 4-figure supplement 3, Source Data 1. Original RT-PCR images corresponding to Figure 4-figure supplement 3A. The left image corresponds to CAL 27. The right image corresponds to SCC-9. *18S* rRNA served as a loading control.
