# Supplementary material for: RUNX2 isoform II protects cancer cells from ferroptosis and apoptosis by promoting PRDX2 expression in oral squamous cell carcinoma
Source: eLife. 2025 Jun 11;13:RP99122. doi: 10.7554/eLife.99122 (PMC12158427; doi:10.7554/eLife.99122)
Supplement: Figure 5—source data 1. [file elife-99122-fig5-data1.zip › Figure 5-Source Data/fig5-source data legends.docx]

**fig5-data1**. PDF file containing original RT-PCR images for Figure 5A, indicating the relevant bands and treatments.

**fig5-data2**. Original files for RT-PCR analysis displayed in Figure 5A.

**fig5-data3**. Original data corresponding to Figure 5A.

**fig5-data4**. Original data corresponding to Figure 5C.

**fig5-data5**. Original data corresponding to Figure 5D.
