# Supplementary material for: RUNX2 isoform II protects cancer cells from ferroptosis and apoptosis by promoting PRDX2 expression in oral squamous cell carcinoma
Source: eLife. 2025 Jun 11;13:RP99122. doi: 10.7554/eLife.99122 (PMC12158427; doi:10.7554/eLife.99122)
Supplement: Figure 5—figure supplement 2—source data 1. [file elife-99122-fig5-figsupp2-data1.zip › Figure 5-figure supplement 2-Source data/fig5-figsupp2-data1.pdf]

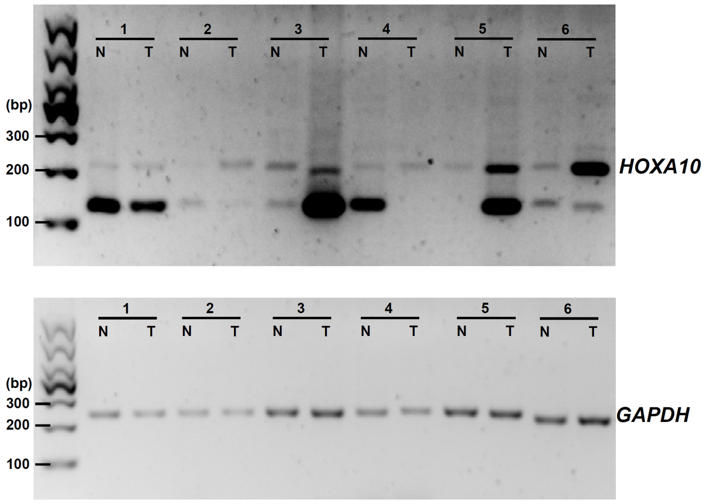

Figure 5-figure supplement 2, Source Data 1. Original RT-PCR images corresponding to Figure 5-figure supplement 2A. *GAPDH* served as a loading control.
