# Supplementary material for: RUNX2 isoform II protects cancer cells from ferroptosis and apoptosis by promoting PRDX2 expression in oral squamous cell carcinoma
Source: eLife. 2025 Jun 11;13:RP99122. doi: 10.7554/eLife.99122 (PMC12158427; doi:10.7554/eLife.99122)
Supplement: Figure 6—source data 1. [file elife-99122-fig6-data1.zip › Figure 6-Source Data/fig6-source data legends.docx]

**fig6-data1**. PDF file containing original RT-PCR images for Figure 6A, indicating the relevant bands and treatments.

**fig6-data2**. Original files for RT-PCR analysis displayed in Figure 6A.

**fig6-data3**. Original data corresponding to Figure 6A.

**fig6-data4**. PDF file containing original western blot images for Figure 6B, indicating the relevant bands and treatments.

**fig6-data5**. Original files for western blot analysis displayed in Figure 6B.

**fig6-data6**. Original data corresponding to Figure 6B.

**fig6-data7**. Original data corresponding to Figure 6C.

**fig6-data8**. Original data corresponding to Figure 6D.

**fig6-data9**. Original data corresponding to Figure 6G.

**fig6-data10**. Original data corresponding to Figure 6H.
