# Supplementary material for: RUNX2 isoform II protects cancer cells from ferroptosis and apoptosis by promoting PRDX2 expression in oral squamous cell carcinoma
Source: eLife. 2025 Jun 11;13:RP99122. doi: 10.7554/eLife.99122 (PMC12158427; doi:10.7554/eLife.99122)
Supplement: Figure 6—figure supplement 1—source data 1. [file elife-99122-fig6-figsupp1-data1.zip › Figure 6-figure supplement 1-Source Data/fig6-figsupp1-source data legends.docx]

**fig6-figsupp1-data1**. PDF file containing original RT-PCR images for Figure 6-figure supplement 1A, indicating the relevant bands and treatments.

**fig6-figsupp1-data2**. Original files for RT-PCR analysis displayed in Figure 6-figure supplement 1A.

**fig6-figsupp1-data3**. Original data corresponding to Figure 6-figure supplement 1A.

**fig6-figsupp1-data4**. PDF file containing original western blot images for Figure 6-figure supplement 1B, indicating the relevant bands and treatments.

**fig6-figsupp1-data5**. Original files for western blot analysis displayed in Figure 6-figure supplement 1B.

**fig6-figsupp1-data6**. Original data corresponding to Figure 6-figure supplement 1B.
