# Supplementary material for: RUNX2 isoform II protects cancer cells from ferroptosis and apoptosis by promoting PRDX2 expression in oral squamous cell carcinoma
Source: eLife. 2025 Jun 11;13:RP99122. doi: 10.7554/eLife.99122 (PMC12158427; doi:10.7554/eLife.99122)
Supplement: Figure 7—source data 1. [file elife-99122-fig7-data1.zip › Figure 7-Source Data/fig7-source data legends.docx]

**fig7-data1**. Original data corresponding to Figure 7B.

**fig7-data2**. Original data corresponding to Figure 7C.

**fig7-data3**. Original data corresponding to Figure 7D.

**fig7-data4**. PDF file containing original RT-PCR image for Figure 7E, indicating the relevant bands and treatments.

**fig7-data5**. Original file for RT-PCR analysis displayed in Figure 7E.

**fig7-data6**. Original data corresponding to Figure 7E.

**fig7-data7**. PDF file containing original western blot images for Figure 7F, indicating the relevant bands and treatments.

**fig7-data8**. Original files for western blot analysis displayed in Figure 7F.

**fig7-data9**. Original data corresponding to Figure 7F.

**fig7-data10**. Original data corresponding to Figure 7G.

**fig7-data11**. Original data corresponding to Figure 7H.
