# Supplementary material for: RUNX2 isoform II protects cancer cells from ferroptosis and apoptosis by promoting PRDX2 expression in oral squamous cell carcinoma
Source: eLife. 2025 Jun 11;13:RP99122. doi: 10.7554/eLife.99122 (PMC12158427; doi:10.7554/eLife.99122)
Supplement: Figure 7—figure supplement 1—source data 1. [file elife-99122-fig7-figsupp1-data1.zip › Figure 7-figure supplement 1-Source Data/fig7-figsupp1-data7.pdf]

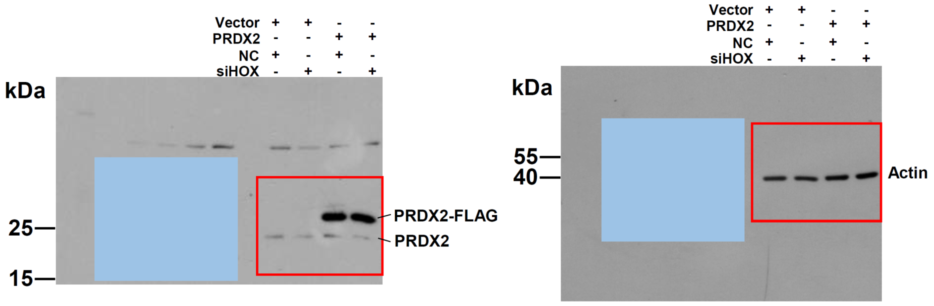

Figure 7-figure supplement 1, Source Data 7. Original Western blot images corresponding to Figure 7-figure supplement 1D. Actin served as a loading control.
