# Supplementary material for: RUNX2 isoform II protects cancer cells from ferroptosis and apoptosis by promoting PRDX2 expression in oral squamous cell carcinoma
Source: eLife. 2025 Jun 11;13:RP99122. doi: 10.7554/eLife.99122 (PMC12158427; doi:10.7554/eLife.99122)
Supplement: Supplementary file 1. [file elife-99122-supp1.docx]

| **Characteristics** | **Number of Cases (%)** |
| --- | --- |
| **Age（Y）** |  |
| <55  ≥55 | 4 (36.4%)  7 (63.6%) |
| **Gender** |  |
| Male  Female | 9 (81.8%)  2 (18.2%) |

**The clinical characteristics of OSCC patients**
