# Supplementary material for: RUNX2 isoform II protects cancer cells from ferroptosis and apoptosis by promoting PRDX2 expression in oral squamous cell carcinoma
Source: eLife. 2025 Jun 11;13:RP99122. doi: 10.7554/eLife.99122 (PMC12158427; doi:10.7554/eLife.99122)
Supplement: Supplementary file 2. [file elife-99122-supp2.docx]

**Primer sequences for RT-PCR**

| **Gene name** | **sequence** |
| --- | --- |
| *RUNX2 isoform II* | F: 5’ GCACAGTGACACCATGTCAGC 3’ |
|  | R: 5’ TGCTGTTGCTGCTGCTGTTG 3’ |
| *RUNX2 isoform I* | F: 5’ TGTGATGCGTATTCCCGTAGATC 3’ |
|  | R: 5’ TGCTGTTGCTGCTGCTGTTG 3’ |
| *PRDX2* | F: 5’ CCAGACGCTTGTCTGAGGAT 3’ |
|  | R: 5’ ACGTTGGGCTTAATCGTGTC 3’ |
| *SOD1* | F: 5’ AAGGCCGTGTGCGTGCTGAA 3’ |
|  | R: 5’ GGCCCACCGTGTTTTCTGGA 3’ |
| *SOD2* | F: 5’ GGAAGCCATCAAACGTGACTT 3’ |
|  | R: 5’ CCCGTTCCTTATTGAAACCAAGC 3’ |
| *CAT1* | F: 5’ CCAGAAGAAAGCGGTCAAGAA 3’ |
|  | R: 5’ GAGATCCGGACTGCACAAAG 3’ |
| *GPX1* | F: 5’ TCGGTGTATGCCTTCTCGG 3’ |
|  | R: 5’ CGTTCTCCTGATGCCCAAA 3’ |
| *HOXA10* | F: 5’ AGAGATTAGCCGCAGCGTCC 3’ |
|  | R: 5’ TTCCTGGGCAGAGCCTGAAG 3’ |
| *GAPDH* | F: 5’ GAAGGTGAAGGTCGGAGTC 3’ |
|  | R: 5’ GAAGATGGTGATGGGATTTC 3’ |
| *Human 18S rRNA* | F: 5’ GGATGCGTGCATTTATCAGA 3’ |
|  | R: 5’ GTTGATAGGGCAGACGTTCG 3’ |
| *TFRC* | F: 5' ACCATTGTCATATACCCGGTTCA 3' |
|  | R: 5' CAATAGCCCAAGTAGCCAATCAT 3' |
| *FIS1* | F: 5' CGGAGCAAGTACAATGATGAC 3' |
|  | R: 5' CCAGGTAGAAGACGTAATCCC 3' |
